# Research into Mercury Exposure and Health Education in Subsistence Fish-Eating Communities of the Amazon Basin: Potential Effects on Public Health Policy

**DOI:** 10.3390/ijerph7093467

**Published:** 2010-09-16

**Authors:** José G. Dórea

**Affiliations:** Department of Nutrition, Universidade de Brasília, P.O. Box 04322, Brasília, DF, 70919-970, Brasil; E-Mail: dorea@rudah.com.br; Tel.: +55-61-3368-3575; Fax: +55-61-3368-5853

**Keywords:** methylmercury, ethylmercury, thimerosal, fish, risk assessment, neurodevelopment

## Abstract

The neurotoxic effects of fish-methylmercury (meHg) consumed regularly are considered hazardous to fetuses and newborn infants; as a result fish consumption advisories are an important asset to control meHg exposure in affluent societies. These concerns are now part of health promotion programs for Amazon subsistence villagers. While urban dwellers in affluent societies can choose an alternative nutritious diet, traditional and subsistence communities are caught up in controversial issues and lifestyle changes with unintended health consequences. Traditional fish-eating populations of industrialized and non-industrialized regions may be exposed to different neurotoxic substances: man-made pollutants and environmentally occurring meHg. Additionally, in non-industrialized countries, pregnant women and infants are still being immunized with thimerosal-containing vaccines (TCVs) which degrade to ethylmercury (etHg). Therefore, the complexity involving fish-meHg associated with wild-fish choices and Hg exposure derived from TCVs is difficult to disentangle and evaluate: are villagers able to distinguish exposure to differently hazardous chemical forms of Hg (inorganic, fish-meHg, and injected etHg)? Is it possible that instead of helping to prevent a plausible (unperceived) fish-meHg associated neurocognitive delay we may inadvertently arouse panic surrounding Hg exposure and disrupt subsistence fish-eating habits (necessary for survival) and life-saving vaccination programs (required by public health authorities)? These questions characterize the incompleteness of information related on the various chemical forms of Hg exposure and the need to convey messages that do not disrupt nutritional balance and disease prevention policies directed at Amazonian subsistence communities.

## 1. Introduction

The neurotoxic effects of regularly consumed fish-methylmercury (meHg) are considered hazardous to fetuses and newborn infants; as such, health concerns have initiated fish advisories in the USA [1] and many other parts of the world. While avoiding high-Hg fish does not constitute a nutrition-specific risk for affluent urban populations, subsistence fish-eating communities cannot replace fish with other nutrition sources. In traditional and subsistence life-styles, fish and seafood are eaten because they are easily caught and recognized as good basic foods, and in urban affluent societies they are recommended because of their proven health benefits. Therefore, for traditional communities, heritage (represented by religion or culture) and survival strategies (socio-economic conditions) make it difficult to avoid environmentally occurring fish-borne substances such as meHg.

Fish is a recognized food item to combat micronutrient deficiencies in developing countries [2], and its importance to the native communities of Rio Madeira has been recently demonstrated [3–5]. The nutritional quality of fish constituents and their role in the health of Amazonians has been reviewed elsewhere [6]. Fish protein is well digested and has a high biological value that complements the dietary energy (70%–80%) derived from starchy (cassava) roots. Fish contains iodine which is important in iodine-depleted soils of tropical rain forests [6]. Additionally, absorption of trace elements (Zn, Fe, I) is enhanced by fish which also contains high levels of Se (known to counteract the toxic effects of Hg) and omega-3 polyunsaturated fatty acids (PUFA; decosahexanoic [22:6] acid (DHA) and eicosapentaenoic [20:5] acid), essential for infant neurodevelopment.

Therefore it is important to distinguish between the adverse effects attributed to meHg in fish and the positive developmental outcomes seen for fish consumption (probably due to micronutrients and especially PUFA) [7]. Indeed, Greiner *et al*. [8] have discussed the role for health communicators in effectively disseminating research about the positive (health benefits of nutrients] and negative (meHg) implications of fish consumption, stressing the impacts of skewed presentations.

## 2. Fish-Targeted Educational Programs in Subsistence Settings

In the specific context of challenges posed by warning vulnerable populations about the possible toxicological hazards of fish-meHg, the international community of environmental-health professionals has been actively engaged in protecting the health integrity of subsistence fish-eating populations. Efforts have been driven by a genuine belief that fish-meHg is a sufficiently great public health concern and that decreasing consumption of certain species should reduce risks related to meHg exposure. Although I do not dispute that certain types of seafood deserve consideration regarding meHg exposure for vulnerable individuals, I do posit that the role of fish consumption for subsistence fish-eating populations is highly complex and requires further investigation. For subsistence riverine populations of the Amazon [6] and North American Tribal communities [9] giving up fish means giving up their main source of nourishment.

Fish-targeted education programs are showing subsistence populations with sufficient knowledge how to prevent mercury contamination, a problem thought by experts to be imminent [10,11]. These health programs assume that the human ecology of subsistence Amazonians has the capacity to maintain their current health status (since there are no perceived consequences arising from their habitual fish consumption, they cannot improve it) while simultaneously absorbing Hg information aiming only to change fish consumption habits. This message about the dangers of mercury *per se* is scientifically sound and well intentioned, but it does not acknowledge the fact that changes in diet may have unintended consequences: especially when the end result is the decrease in fish consumption or worse, replacing fish with foods that are less healthy [12].

While the concept of cutting fish consumption is emerging as inadequate to supply essential nutrients [13] and to maintain good health in affluent societies [14] it imposes an unfair burden on traditional populations [9]. Harper and Harris [9] have elegantly addressed equivocal policies that rely solely on fish-consumption advisories for native populations; they argue that such policies shift the burden of avoiding risk to the very people burdened by contaminant exposure. Harper and Harris [9] classify this situation as environmental injustice: “*Chemical contamination places Tribes in a lose-lose situation: either eat the fish and suffer the health effects from contaminants*, *or do not eat the fish and suffer the health and cultural effects of lost fish*”.

Unlike North American Tribal communities, Amazonian subsistence villagers are threatened by far worse health problems related to nutrition (anemia) and infectious diseases (diarrhea, malaria, dengue, yellow fever) than by unperceived neuro-behavioral deficits attributed to fish-meHg. Indeed, Fillion *et al*. [15] showed that despite elevated Hg exposure, Amazonian riverines (13 villages), reported perceptions of quality of life and health, associated mainly with the absence of chronic illnesses. Recently, Muniz *et al*. [16] reported that anemia and intestinal parasites are still a public health problem for Amazonian children.

## 3. Persistent Bioaccumulative Substances: Biomarkers of Fish Consumption and Neurotoxic Exposure

Persistent substances tend to accumulate in fish [17] and, recent studies have indicated a strong, direct and independent relationship between fish consumption and bio-accumulative substances (pollutants and essential nutrients) in tissues of consumers [18,19]. Indeed, traditional cultures in other parts of the American continent are exposed to various chemicals that bioacccumulate in fish and are also neurotoxic [20]. Because organohalogens have been found in Amazonian fish [21], it does not come as a surprise that subsistence populations may also be exposed to neurotoxic substances other than meHg.

In a broader scientific context, the purported neurological deficits attributed to hazardous substances in fish have been related not only to meHg but to organohalogenated pollutants [17]. Because of near-wilderness conditions and the absence of industrial activities, Amazonian high fish-eaters are likely to be exposed to attendant geochemically occurring meHg, but much less to other pollutants occurring in industrialized regions. Differently from Amazonian villagers (known as “*ribeirinhos*”), Native Americans of the Great Lakes and Canada are exposed to both meHg and organohalogenated pollutants from industrial activities [22].

In subsistence villagers of the Amazon Basin, mercury contamination is indeed derived from fish-meHg, a correlation supported by the fact that hair-Hg concentrations along hair strands reflect variation in fish consumption due to seasonal high and low waters [23]. Indeed, it is possible that the fall of 35% in hair mercury can be accounted for by changing relative proportions of piscivorous for herbivorous fish—which contain much less meHg [11]. However, fish-meHg was first thought to be a direct result of gold mining activities; lately, geochemical mercury release into the aquatic ecosystems of the Amazon Basin is believed by some to increase as the consequence of growing deforestation rates and the rapid expansion of agricultural activities [11]. These activities may also release agriculture pesticides and other bioaccumulative and neurotoxic substances. Additionally, other unsuspected sources of neurotoxic substances may arise from activities unrelated to fish but central to subsistence living. Recently it has been found that in some communities of the Eastern Amazon the metal pans used in the final stage of manioc-flour preparation are suspected of being a source of Pb contamination, and that this is responsible for high Pb blood levels in these communities [24]. Despite that, it is believed that “the populations exposed to meHg through fish consumption are placed at a disadvantage with respect to adequate development of intellectual and physical capacities because of nervous system deficits” [11].

## 4. Fish Consumption and Neurodevelopment in the Amazon

Fish is widely consumed in the Amazon region. When hair-Hg concentrations are used as biomarkers of fish consumption, studies show a wide range in Amazonian populations [25]. Because of non-invasive characteristics and convenience of storage and transportation, hair-Hg is the preferred tissue to study both meHg exposure and fish consumption.

Some studies have found deficits in neuro-functional tests among Amazonian adults [26] and children [27]. Passos and Mergler [27] summarized neurobehavioral studies in children: mean HHg levels (>10 ppm) tended to show a significant decrease in tested neurobehavioral functions, while other studies in children with lower mean HHg (<5 ppm) showed no significant effects [28]. Indeed, neurofunctional testing of isolated communities could be related to causes other than fish consumption; we [29] have showed that riverine children with fish-meHg exposure (hair-Hg concentrations) 66 times higher than that found in isolated rural agrarian communities of South-East Brazil had equally poor neurofunctional outcomes. In recent years we have monitored the nutritional transitioning of subsistence living for some traditional riverine populations, charting a substantial decline in fish consumption (measured as hair-Hg) and no apparent benefit in neurodevelopment tests [30].

Chevrier *et al*. [31] compared Amazonian fish-eating populations (Wayama children of French Guiana *versus* Brazilian children of Rio Tapajós); there were increased risks of making rotation or simplification errors in the drawings by Brazilian children with increased Hg exposure. These children had twice the mean levels of hair-Hg concentrations than the Wayama children (French Guiana). Besides a higher mean fish intake, Brazilian children are additionally exposed to ethylmercury (etHg) in thimerosal-containing vaccines (TCV) during gestation and infancy. Furthermore, a *post hoc* discussion disclosed that, unlike the French immunization schedule used in French Guiana, the Brazilian Hepatitis B vaccination starts much earlier—at birth [32]. Indeed, Amazonian infants are sensitive to in utero mercury exposure (which also includes vaccine-etHg) as measured by neurodevelopment tests at six months [33]. It is worth mentioning that breastfed children are capable of overcoming early mercury sensitivity (as measured by neurobehavioral testes) by the age of 60 months [34]. Indeed, neurofunctional outcomes in fish consumers can be modulated by home environment [35] and can respond to other neurotoxic substances found in fish. Plusquellec *et al*. [20] assessed child behavior as a function of exposure to polychlorinated biphenyls (PCBs), lead (Pb), and mercury (Hg) in Inuit children reporting that only cord and child blood-Hg concentrations were not related to any tested behavioral outcomes. Indeed, moderately high intakes of fish consumption during pregnancy have been suggested as beneficial for neurodevelopment of children [36].

## 5. Contextualizing Mercury Exposure and Risks of Neurodevelopment

As far as organic forms of Hg are concerned, Amazonians (in Brazil and perhaps in most other countries crossed by the river) are additionally exposed to TCV. Therefore, there are emerging sensitive issues in the broader picture of all forms of organic Hg exposure that need special attention. Infants’ exposure to organic mercury is also iatrogenic, coming from sources such as TCVs [37] as thimerosal used in these vaccines degrades to etHg. Despite that, TCVs are widely used in Brazil in pregnant women [33] and in young children [37] and are associated with transient neurodevelopment delays [33,34,38]. Although TCVs are considered safe, when confronted with the plausibility of subtle neurodevelopment deficits, some epidemiological studies have found both positive and negative effects that were attributed to chance [39]. There are, however, epidemiological studies that point to significant links between exposure to TCVs and neurodevelopmental delays [39].

Health education projects in the Amazon Basin villages have been successful in raising community interest in Hg and human health; it seems that mercury contamination is now an important issue for Rio Moroni [10] and Rio Tapajós [11] villagers. At the same time, the vaccine coverage of the Brazilian Amazon’s population has increased significantly as a result of the Brazilian government’s public health policies. In the present Brazilian vaccination calendar, 90% of children are immunized with TCVs (hepatitis B, and DTP). Exposure to etHg through TCV happens before and after birth in these populations [33,37]. In countries still using these vaccines, children’s hair-Hg (accepted as marker of fish consumption) has been shown to increase after immunization [40–42]. Indeed, etHg has been found in hair of infants after immunization with TCVs [43]. Only recently, research (*in vitro* and animal studies) showed toxicity effects compatible with low-dose-mercury but as yet without a clear clinical significance [44]. Sooner or later, this population will be also exposed to the current controversy of etHg in infants’ vaccines and risk of neurodevelopment delay. Indeed, *Ciência Hoje*, a popular science magazine, recently featured this controversial issue on its cover [45].

## 6. Nutrition and Food-related Toxins in the Human Ecology of Subsistence Communities

The environmental health workers in villages of the Amazon are to be commended for promoting community awareness of environmental contaminants. However, it is important to emphasize that both cassava and fish, the two most important staple foods in the *ribeirinhos*’ diet, contain respectively linamarin and meHg; these are recognized neurotoxins. In parts of Africa, where residual cyanide (from unprocessed cassava) and poor-protein diets combine to cause “konzo” (a disabling neuropathy), cassava-linamarin is a public health issue. Linamarin present in unprocessed cassava is properly destroyed before consumption by processing methods used by native Amer-Indians [46]; small intakes of residual cyanogenic glycoside can be effectively metabolized by the high intake of fish that carries protein-containing sulfur amino acids [47]. Therefore, aggravation of iodine deficiency disorders (due to cassava consumption) and susceptibility to “konzo” seem to be associated with under-nutrition or lack of essential nutrients that in Amazonia are provided by fish [6]. Indeed, in the last two decades, neuropathy outbreaks associated with food/nutrition have not been related to fish-eating habits [47]: the neuropathy outbreak in Cuba involved poor nutrition, whereas endemic “konzo” is associated with residual cassava cyanogens. So far, Amazonian *ribeirinhos* have shown no predisposition for degenerative CNS diseases associated with lifetime consumption of naturally occurring toxins in either cassava or fish.

Indeed, the nutritional status of Amazonian settlers is poorer than adapted Amer-Indians [48]. And, among Amer-Indians, those eating more fish appear to have a better nutritional status [5] and better cardiovascular health [49]. However, cardiovascular benefits of fish consumption and attendant risk of meHg on carviovascular health may have modifying factors that are not yet fully understood. While for some population fish-Hg may increase the risk of cardiovascular diseases [50], for others it may not [51]. Indeed it appears that DHA could counteract negative effects of meHg resulting from fish consumption [52].

Although we need to work on preventive/interventional strategies that can help subsistence populations, we need more research to clarify what we are preventing: which neurologic or health risks are present as a result of consuming fish. Education programs that target fish species with high meHg concentrations may not be effective in certain Amazonian populations. Cerdeira *et al*. [53] observed that *ribeirinhos* (riverines) that traded their catch concentrated on smaller species for local consumption. Therefore, risk assessment based on specific fish species (usually large predators) or mean Hg concentrations [54] may not be suitable for subsistence populations of the Amazon Basin; Passos *et al*. [54] have found other dietary items that might interfere with risk prediction assessment.

Indeed, fish consumption is part of a successful strategy for survival and should be considered as a health asset. Specifically in the Amazon’s riverine populations, despite high body Hg load, we have shown that clinical evaluation did not detect symptoms compatible with mercury toxicity (paraparesis, tremor, numbness of limbs, sensory disturbances), but there was a high (71%) incidence of clinical history of malaria [55]. Furthermore, subsistence populations of the Amazon often do not have the choice of giving up fish. For some populations fishing is a source of income and for more traditional groups fish represent also their culture, heritage and religion, which are intimately connected with certain animals and food-cycles [9]. Meanwhile, we should also strive to have vaccines that do not increase the Hg burden on infants and children: currently the first line of organic-Hg exposure (immunization with TCV).

## 7. Conclusions

Summing up, traditional fish-eating populations of industrialized and non-industrialized regions may be exposed to different neurotoxic substances. Despite that, the complexity involving fish-meHg associated with wild fishing choices and Hg exposure derived from TCVs (in countries that use these vaccines) makes it difficult to discern, disentangle and evaluate complex and crucial issues: are villagers able to distinguish exposure to hazardous chemical forms of Hg (inorganic, fish-meHg, and injected TCV-etHg)? Is it possible that instead of helping to prevent a plausible (unperceived) harm we may inadvertently arouse panic surrounding Hg exposure and disrupt subsistence fish eating and a very successful vaccination program? An approach aiming to minimize mercury exposure of these populations requires more than taxa-related mercury concentrations; it demands integrated social actions involving community lifestyle, public health policies and eco-health research. First, we have to follow the path of developed nations and adopt thimerosal-free vaccines for pregnant mothers and young children; until then, while translating research of low-dose TCV-etHg, it is essential to preserve the trust these populations have put in vaccines to prevent infectious diseases that exacerbate the health inequalities of these communities. Therefore, it is crucial for educational programs that might change fish eating habits to consider the nutritional benefits and health effects attendant on fish consumption in subsistence communities.
